# Propuesta Para un Uso Responsable de la Inteligencia Artificial Generativa en la Práctica Médica

**DOI:** 10.31083/RN37503

**Published:** 2025-08-27

**Authors:** David A. Pérez Martínez

**Affiliations:** ^1^Servicio de Neurología, Hospital Universitario 12 de Octubre, 28041 Madrid, Español; ^2^Servicio de Neurología, Hospital Universitario La Luz, 28003 Madrid, Español

**Keywords:** inteligencia artificial, ética médica, prestación de atención sanitaria, investigación biomédica, educación médica, práctica clínica, artificial intelligence, medical ethics, delivery of health care, biomedical research, medical education, clinical practice

## Abstract

**Introduccion:**

El avance de la inteligencia artificial (IA), especialmente la IA generativa, ha revolucionado la medicina impactando en la asistencia sanitaria, docencia e investigación. Si bien las oportunidades son numerosas, la implementación de la IA plantea desafíos éticos y técnicos, como el riesgo derivado del sesgo en los datos, la posible pérdida de habilidades clínicas o los relacionados con la privacidad de la información.

**Desarrollo:**

La IA ha demostrado su capacidad para optimizar procesos médicos y educativos. Sin embargo, su funcionamiento basado en la predicción probabilística está sujeto a errores y sesgos. El profesional debe conocer estos riesgos y abogar por una implementación transparente, responsable y segura, manteniendo la responsabilidad legal y ética de las decisiones clínicas. Hay que ser vigilantes en la preservación de las habilidades clínicas profesionales, abogando por un uso prioritario de la IA en la eliminación de actividades automatizadas de bajo valor añadido. En la investigación biomédica, la transparencia y la validación independiente son esenciales para garantizar resultados reproducibles. Igualmente, en la formación médica, es clave que los profesionales reciban una educación estructurada en IA para que puedan integrarla en su actividad clínica de forma segura.

**Conclusiones:**

La IA generativa ofrece un potencial transformador para la medicina, pero requiere de un enfoque riguroso y ético. La formación integral, la mitigación de riesgos y la preservación de habilidades clínicas tradicionales son pilares para su adopción responsable. Este cambio debe ser liderado desde la profesión médica promoviendo una medicina centrada en el paciente.

## Abstract (en)

**Introduction:**

The advancement of artificial intelligence (AI), particularly generative AI, has significantly transformed the field of medicine, impacting healthcare delivery, medical education, and research. While the opportunities are substantial, the implementation of AI also raises important ethical and technical challenges, including risks related to data bias, the potential erosion of clinical skills, and concerns about information privacy.

**Development:**

AI has demonstrated great potential in optimizing both clinical and educational processes. However, its operation based on probabilistic prediction is inherently prone to errors and biases. Healthcare professionals must be aware of these limitations and advocate for a transparent, responsible, and safe integration of AI, while maintaining full ethical and legal responsibility for clinical decisions. It is essential to safeguard traditional clinical competencies and prioritize the use of AI in automating low-value, repetitive tasks. In biomedical research, transparency and independent validation are crucial to ensure the reproducibility of findings. Similarly, in medical education, structured training in AI is vital to enable professionals to apply these tools safely and effectively in clinical practice.

**Conclusions:**

Generative AI offers a transformative potential for medicine, but its adoption must be guided by rigorous ethical standards. Comprehensive training, risk mitigation, and the preservation of core clinical skills are essential pillars for its responsible implementation. This transformation must be led by the medical profession to ensure a patient-centered approach to care.

## 1. Introduccion

La inteligencia artificial (IA) está transformando las ciencias de la salud, no solo en la asistencia sanitaria, sino también en los campos de la docencia y la investigación médica. Su capacidad para procesar grandes volúmenes de datos y generar conocimiento está redefiniendo la forma tradicional de trabajar. Sin embargo, junto con estas oportunidades, emergen retos éticos, técnicos y sociales que exigen un enfoque responsable para garantizar que la IA cumpla con criterios de seguridad y equidad. Su uso se ha extendido en todos los ámbitos. En el reciente informe “*AI Adoption in Healthcare Report 2024*”, elaborado por la *Healthcare Information and Management Systems Society* (HIMSS) y el portal de formación sanitaria *Medscape*, hasta el 50% de los médicos en ejercicio utilizan herramientas de IA al menos de forma ocasional. Este uso está principalmente orientado a tareas como búsqueda y análisis de literatura médica, apoyo en el diagnóstico, y planificación de tratamientos [1].

Los potenciales riesgos asociados al uso de la IA en la asistencia sanitaria incluyen la presencia de sesgos en los datos de entrenamiento, la posibilidad de una degradación de las habilidades clínicas tradicionales, las dudas en relación con la privacidad de los datos sanitarios o la falta de transparencia en su uso. En esa línea, la IA genera inquietud al no quedar clara la responsabilidad del profesional sanitario en las decisiones tomadas con su ayuda, lo que agrava la inseguridad jurídica y plantea dudas sobre las implicaciones éticas y legales en la práctica médica. En el campo de la docencia, es clave que los futuros profesionales puedan entender y supervisar estas tecnologías de manera crítica. Sin embargo, esta necesidad se contrapone al peligro de una deshumanización de la relación médico-paciente derivada de una enseñanza centrada excesivamente en herramientas tecnológicas en detrimento del cultivo de las habilidades interpersonales. Por último, y no menos importante, las oportunidades de la IA en el ámbito de la investigación médica son múltiples y abren posibilidades sin precedentes para analizar grandes bases de datos y generar hipótesis innovadoras. Además, supone una herramienta de gran ayuda a la hora de generar contenidos para todos los ámbitos, aunque plantea el dilema sobre hasta qué punto se define la autoría de los trabajos realizados con su ayuda y la responsabilidad sobre los mismos una vez publicados.

Como vemos, las oportunidades y riesgos que plantea la IA son múltiples, y su impacto dependerá de cómo afrontemos los retos actuales. El futuro de la IA en medicina no está predeterminado, será moldeado por las decisiones que tomemos hoy. Es imperativo que sean los profesionales sanitarios, en estrecha colaboración con los pacientes, quienes asuman el liderazgo en la integración de la IA en la práctica clínica. Las administraciones y desarrolladores tecnológicos, si bien tienen un papel importante, deberían mantener un rol consultivo y de apoyo. Tenemos el riesgo de repetir el modelo de transformación digital sanitario iniciado en los años 90 en el sistema sanitario. La implementación de la historia clínica electrónica ha recibido numerosas críticas, en gran medida debido a una falta de liderazgo profesional durante el proceso y a centrar excesivamente la atención en una interacción con los sistemas informáticos, en vez de favorecer la comunicación médico-paciente [2, 3].

En cualquier caso, la IA ha llegado para imponer un nuevo paradigma en todos los ámbitos del conocimiento y su empleo se ha difundido por toda la sociedad. Una de las herramientas más populares de IA generativa, *ChatGPT* de *OpenAI*, se lanzó el 30 de noviembre de 2022 y tan solo cinco días más tarde, el 4 de diciembre, se estimaba que ya tenía más de un millón de usuarios [4]. Actualmente se calcula que hay más de 300 millones de usuarios habituales de esta herramienta en el mundo [5]. Y en un futuro cercano será imposible saber el número de personas que emplean estas herramientas, ya que la esencia de los modelos de IA estarán embebidos en todos los sistemas tecnológicos y de comunicación. En sus inicios, Internet se entendía como una herramienta para buscar información o visitar páginas en la web. Sin embargo, evolucionó hacia un sistema global que sustenta actualmente las comunicaciones, el comercio, la logística y las aplicaciones esenciales de toda la sociedad. Hoy, Internet es la infraestructura invisible que conecta y mantiene en funcionamiento prácticamente cualquier sistema complejo. De manera análoga, ahora la IA puede entenderse como un conjunto de herramientas puntuales, como pueden ser los *chatbots*, la generación de contenidos o los sistemas inteligentes de análisis y generación de imágenes o video [5, 6]. Esta visión no refleja su verdadero potencial. En el futuro, la IA estará integrada en todos los sectores sociales y económicos, desde la optimización de la logística global a la medicina personalizada, pasando por la gestión financiera y la sostenibilidad ambiental. Así como Internet transformó la comunicación y los sistemas globales, la IA será la infraestructura cognitiva que potenciará y conectará todas las actividades humanas.

Este trabajo se plantea como una revisión narrativa enfocada en el uso responsable de la IA en el campo de las ciencias de la salud, centrándonos específicamente en la IA generativa. Esta rama de la IA se ha especializado en crear contenido original, como texto, imágenes o datos, a partir de modelos entrenados, diferenciándose de otras formas de IA centradas en tareas de clasificación, predicción o reconocimiento. La mayor parte de esta revisión usaremos el concepto de IA como sinónimo de IA generativa orientado a la generación de contenidos. En cualquier caso, el empleo de esta tecnología debe partir de la necesidad de un análisis de los riesgos y oportunidades. Para ello es clave abordar principios fundamentales como la equidad, transparencia, seguridad y privacidad de los datos. También se debe priorizar la capacitación ética y técnica de los profesionales sanitarios, investigadores y educadores para comprender los límites de la IA. En todos los casos, es fundamental que los profesionales y las organizaciones empleen estas herramientas con transparencia, manteniendo la responsabilidad ética y legal en las decisiones tomadas con su ayuda. El desarrollo de la IA en medicina no puede depender únicamente del entusiasmo y la fascinación que ejerce la tecnología. Se requiere un enfoque deliberado para asegurar que estas herramientas se utilicen poniendo a la persona que recibe el servicio en el centro del sistema. El autor propone una serie de directrices que puedan servir de punto de partida en el desarrollo de ese marco de uso responsable (Tabla 1). No obstante, también es importante subrayar las limitaciones de este análisis teniendo en cuenta el escenario dinámico e incierto en el que se mueven estas tecnologías en el momento actual. Los riesgos y oportunidades actuales podrían ser minimizados o exacerbados en los próximos años dependiendo de la aparición de innovaciones tecnológicas.

**Tabla 1.** **Propuesta de manifiesto para un uso responsable de la inteligencia artificial (IA) en ciencias de la salud**.

**1. La IA debe servir de apoyo en las decisiones clínicas, nunca reemplazarlas, aportando respuestas justificadas o explicativas**. La inteligencia artificial debe complementar el juicio clínico y la relación médico-paciente, y nunca sustituirlos sin supervisión. Hay que apoyar el desarrollo de modelos de IA explicativos que justifiquen las decisiones tomadas, siendo vigilantes ante el riesgo de sesgos en su entrenamiento.
**2. Es imprescindible velar por preservar las habilidades clínicas tradicionales en un escenario de difusión de la IA**. Es esencial evitar que la dependencia de la IA debilite la capacidad de los médicos para tomar decisiones autónomas y reduzca sus habilidades clínicas.
**3. Se debe mantener la responsabilidad médica en todas las decisiones clínicas, independientemente del apoyo de la IA**. El médico debe mantener la responsabilidad ética y legal en las decisiones clínicas, sin delegarla en la IA.
**4. Se debe exigir las mismas evidencias científicas en el empleo de la IA que en otras innovaciones del campo biomédico**. Las herramientas de IA deben cumplir con criterios estrictos de transparencia, reproducibilidad y validez científica, como cualquier otra intervención médica o innovación sanitaria.
**5. Se debe dar prioridad en el uso de la IA para la eliminación de las tareas automatizadas y de bajo valor para el médico**. La IA debe priorizar su uso en ahorrar tiempo en tareas de bajo valor, reservando al médico para actividades fundamentales que mejoren la humanización y la comunicación.
**6. La IA debe integrarse de forma transparente para pacientes y profesionales**. Los usuarios deben saber si interactúan con un sistema de IA o un humano y conocer el grado de participación de la IA en su asistencia. Los profesionales deben declarar el uso de IA en materiales desarrollados para asistencia, docencia e investigación, asumiendo siempre la responsabilidad del contenido.
**7. Debe existir una protección adecuada de los datos privados al emplear IA**. Es fundamental en asistencia e investigación clínica garantizar la privacidad de los datos en el uso de IA, reflejando dicha protección en el consentimiento informado establecido con pacientes y sujetos de estudios.
**8. Hay que estar vigilantes ante los potenciales sesgos de los sistemas de IA**. Los investigadores deben identificar y mitigar los sesgos en los sistemas de IA, promoviendo la reproducibilidad de los resultados con diferentes herramientas, contextos y grupos.
**9. Los médicos debemos disponer de una formación integral y transversal en IA**. Es necesario incorporar una formación sólida sobre IA en todas las etapas de la educación médica, incluyendo las etapas de pregrado y posgrado.
**10. Hay que apoyar la colaboración institucional en la formación sobre IA**. Las administraciones públicas y sociedades científicas deben trabajar juntas para crear un camino formativo integral que oriente el uso adecuado de la IA en medicina.

## 2. Desarrollo

### Qué es la Inteligencia Artificial

La IA es una disciplina que ha evolucionado significativamente desde su origen en la década de 1950, cuando el término fue acuñado por John McCarthy [7, 8]. Definida inicialmente como “la ciencia y la ingeniería de crear máquinas inteligentes”, la IA busca replicar habilidades humanas como el razonamiento, la resolución de problemas y el aprendizaje. El desarrollo de la IA comenzó con sistemas rudimentarios que buscaban emular decisiones humanas. En la década de 1970, aparecieron las primeras aplicaciones médicas, como INTERNIST-1, un sistema de consulta médica, y MYCIN, diseñado para ayudar en la prescripción de antibióticos [9].

**Fig. 1.**
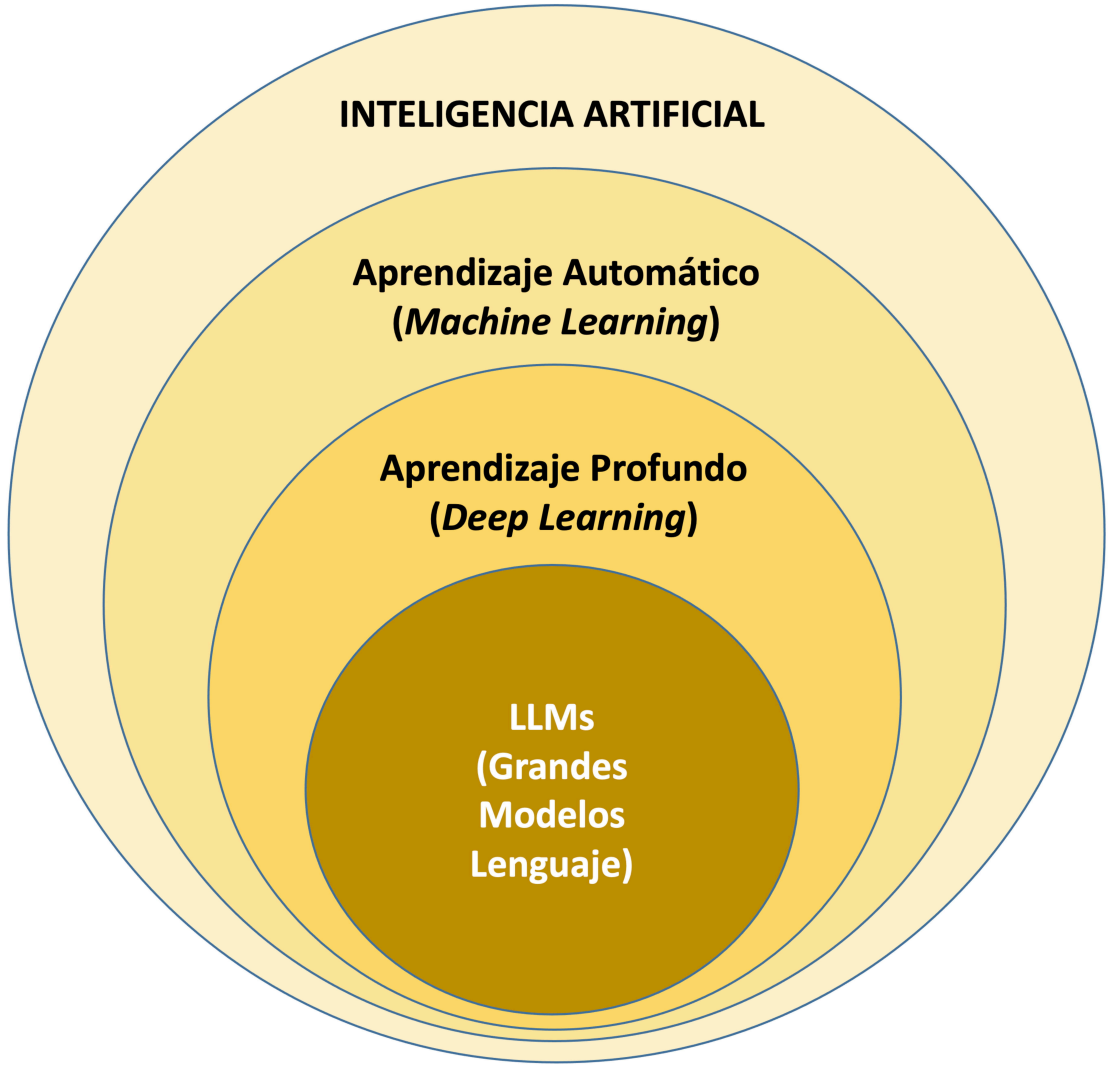
**Diagrama conceptual de la Inteligencia Artificial y los distintos desarrollos tecnológicos que engloba bajo este concepto**.

Hoy en día, entendemos la IA como el desarrollo de máquinas que son capaces de realizar tareas que usualmente requieren de una inteligencia humana. Aunque no existe una definición única de lo que constituye la inteligencia, un consenso clásico de expertos pretendió definirla como el conjunto de capacidades humanas que incluye el razonamiento, la planificación, la resolución de problemas, el pensamiento abstracto, la capacidad de comprender conceptos complejos y aprender de la experiencia previa [10]. En sus inicios, la IA basó su desarrollo en enfoques deterministas, como sistemas de reglas y diagramas de flujo, que estaban diseñados para guiar en decisiones específicas. Se trataba de métodos rígidos y propensos a errores cuando se enfrentaban a escenarios dinámicos. Posteriormente, se desarrollaron métodos más avanzados como el aprendizaje automático (*machine learning*) y el aprendizaje profundo (*deep learning*). Estas tecnologías, menos rígidas y más flexibles, permiten procesar grandes volúmenes de datos, ajustando sus respuestas de manera autónoma y aprendiendo de las experiencias acumuladas durante este proceso. El aprendizaje automático o *machine learning*, una rama clave de la IA, se centra en diseñar sistemas que identifiquen patrones y relaciones entre los datos. Esto permite mejorar progresivamente su desempeño sin que sea necesario una intervención humana directa. Los algoritmos involucrados en estas tareas engloban desde métodos estadísticos clásicos, como la regresión lineal, hasta complejas redes neuronales que han impulsado los avances recientes en este campo (Fig. 1).

Toda esta generación de conocimiento ha desembocado en lo que conocemos actualmente como los grandes modelos de lenguaje (LLMs, por sus siglas en inglés), que representan uno de los avances más significativos en la IA. Los LLMs representan el tipo más popular de IA generativa. Se denominan así al estar diseñados para generar contenido nuevo basado en los datos con los que fueron entrenados. Estos modelos se basan en arquitecturas avanzadas de aprendizaje profundo o *deep learning* como las redes neuronales transformadoras. Este modelo, conocido como *transformers*, fue introducido en la IA tras la publicación de un artículo clave titulado “*Attention is all you need*”, desarrollado por el equipo de investigación de *Google* [11]. Esta innovación ha sido fundamental para entender el estado de desarrollo actual de los LLMs. Su funcionamiento se basa en el concepto de atención y, en particular, en la atención auto-regresiva. De esta forma, el modelo evalúa todas las palabras de una secuencia en paralelo y determina cuáles son más relevantes entre sí en cada contexto. Además, a diferencia de los sistemas previos, los LLMs son entrenados empleando enormes cantidades de datos provenientes de múltiples fuentes como libros, artículos científicos e información publicada en páginas web, lo que les permite aprender patrones complejos del lenguaje humano. El funcionamiento básico de un LLM consiste en procesar texto y generar respuestas mediante la predicción probabilística de las palabras más adecuadas en un contexto dado. Esto les otorga una serie de capacidades que no habían sido observadas previamente, como generar texto coherente, responder preguntas, traducir idiomas y realizar tareas relacionadas con el lenguaje natural. Todo ello ha desembocado en un hito histórico en la interacción entre humanos y máquinas [12]. Hay que añadir que la mayoría de las aplicaciones actuales en este campo se están realizando con el uso de este tipo de IA generativa. Este concepto define una rama específica de la IA centrada en la creación de nuevos contenidos, como texto, imágenes, audio o código a partir de patrones aprendidos durante su entrenamiento. A diferencia de otros sistemas de IA, que suelen limitarse a clasificar, predecir o reconocer datos, la IA generativa tiene la capacidad de producir información original, simulando la creatividad humana dentro de unos márgenes probabilísticos definidos. Aunque con frecuencia se emplea el término LLM como sinónimo de IA generativa, es importante precisar que los grandes modelos de lenguaje constituyen solo una categoría dentro de este campo. Dicho de otro modo, todos los LLM son IA generativa, pero no toda la IA generativa se basa en LLM. No obstante, debido a su versatilidad y amplio uso actual, especialmente en tareas de generación de texto e imagen, los LLM se han convertido en la herramienta más representativa de esta tecnología en la práctica clínica, educativa e investigadora.

## 3. La Inteligencia Artificial en la Asistencia Sanitaria

La incorporación de la IA en la práctica médica representa una oportunidad para optimizar el diagnóstico, tratamiento y gestión de los pacientes. Sin embargo, su implementación debe estar guiada por principios claros que prioricen la ética, la seguridad y la relación médico-paciente. La IA debe concebirse como una herramienta de apoyo diseñada para complementar, pero nunca sustituir, el juicio clínico. Este equilibrio es esencial para preservar la relación médico-paciente, que constituye el núcleo de la práctica médica, fundamentada en la empatía, la confianza mutua y el análisis personalizado de cada caso. Es imperativo adoptar una postura vigilante frente al riesgo de que la IA desplace o debilite las habilidades clínicas esenciales de los profesionales sanitarios. Hay que subrayar que la experiencia y la capacidad de tomar decisiones de forma independiente son los pilares fundamentales de la medicina, y su erosión podría comprometer tanto la calidad de la atención como la seguridad del paciente [13]. En este contexto, la responsabilidad ética y legal de las decisiones clínicas deben permanecer exclusivamente en manos del médico. El uso de algoritmos o aplicaciones basadas en IA no debería conducir a una delegación de la responsabilidad profesional. El médico debe ser el garante final de las decisiones diagnósticas y terapéuticas, asegurando que cada intervención esté orientada por el principio de beneficencia para el paciente. Todo ello requiere que el profesional mantenga un control consciente y fundamentado sobre las recomendaciones emitidas desde las herramientas de IA.

**Fig. 2.**
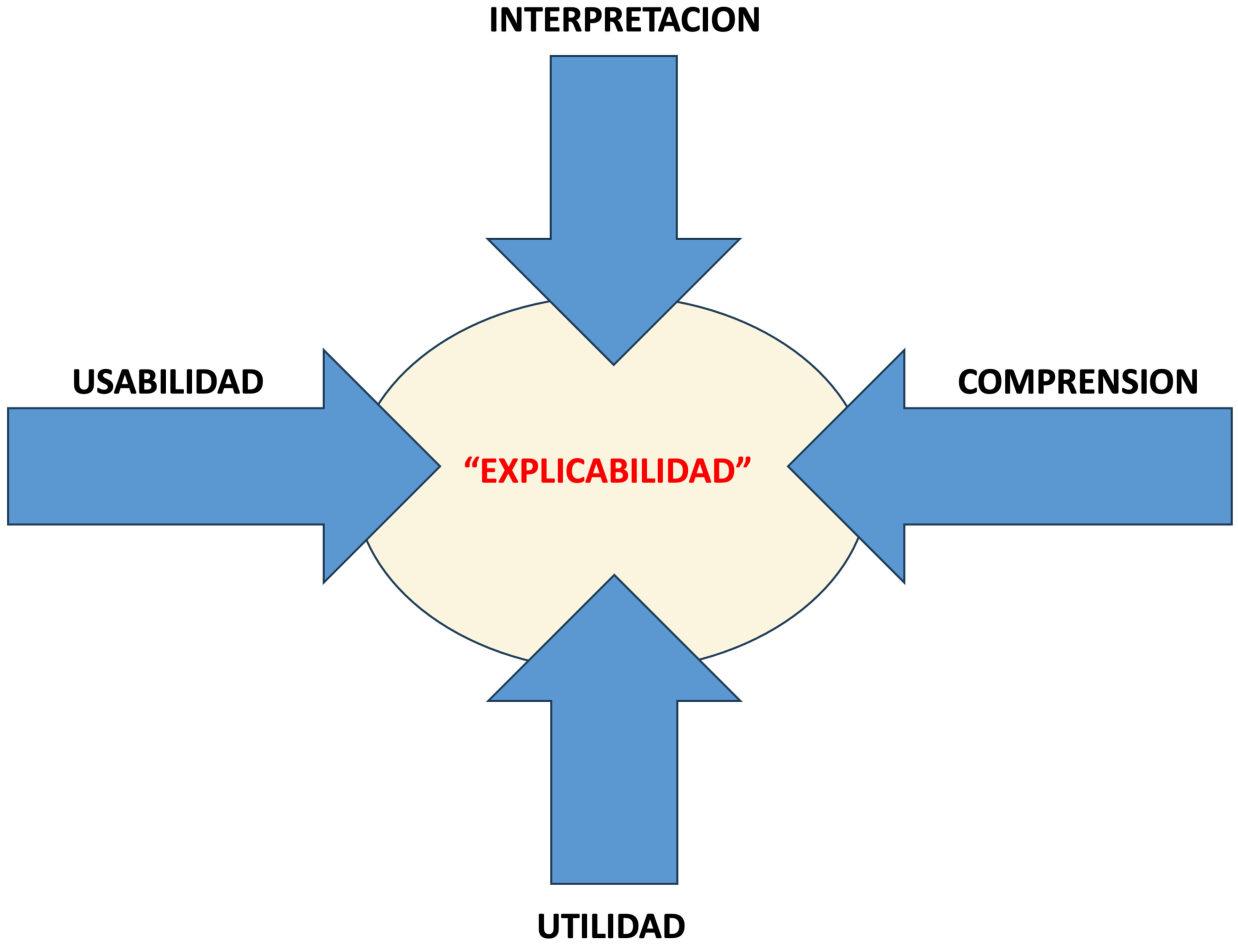
**Esquema representativo de los elementos que pueden ayudar a buscar la justificación o “explicabilidad” de las respuestas propuestas por los modelos de inteligencia artificial**. La explicación de la respuesta debe estar basada en los cuatro elementos propuestos: la interpretación de los resultados, la comprensión del modelo por parte del profesional, la usabilidad del sistema y, finalmente, la utilidad de la respuesta en el contexto clínico [14].

Una limitación de los sistemas complejos de IA es la dificultad para encontrar una explicación evidente de los resultados obtenidos por estás técnicas. Habitualmente, la IA se comporta como una “caja negra” obligando a aceptar sus respuestas sin una justificación explícita. Se entiende como “explicabilidad” a la capacidad de una IA para clarificar sus decisiones. No siempre es sencillo obtener explicaciones claras de los sistemas de IA, ya que procesan grandes volúmenes de datos mediante algoritmos complejos. Estas operaciones incluyen desde cálculos matemáticos sencillos hasta modelos avanzados basados en redes neuronales, lo que dificulta entender cómo llegan a una conclusión específica. Sin embargo, la “explicabilidad” resulta fundamental en el contexto sanitario ya que aporta seguridad clínica y confianza al profesional sanitario que recibe dicha información. Para abordar esta problemática se ha propuesto un modelo multidimensional que combine la interpretación, comprensión, usabilidad y utilidad de los resultados aportados por la IA (Fig. 2, Ref. [14]). Respecto a la capacidad de interpretación, la entendemos como el grado en el que el profesional puede intuir cual es la causa de una decisión tomada por la IA. En términos clínicos, esto puede significar que un médico pueda comprender cómo ciertos datos de entrada llevaron a una predicción específica y, en cierta manera, esperable por su experiencia previa. El grado de comprensión del resultado está asociado al conocimiento que tiene el profesional sobre el funcionamiento de los modelos de IA empleados. Esto incluye los riesgos determinados por el malfuncionamiento del sistema y los errores imprevistos. También debe incluir un conocimiento del tipo de datos empleados en el entrenamiento del modelo, y del tipo de información externa al que el modelo puede acceder en tiempo real. La usabilidad es una característica bien conocida en los sistemas informáticos y asocia la facilidad con la que un usuario interacciona con el sistema. En este contexto, la usabilidad asegura la confianza de los profesionales con un modelo accesible y sencillo. Finalmente, la utilidad mide el grado en el que el sistema satisface las necesidades del usuario y resuelve el problema planteado en la práctica clínica. Bajo este modelo propuesto, hay que integrar a profesionales, pacientes y técnicos en el desarrollo de una IA con “explicabilidad”. En todo caso, probablemente, sin un apoyo explícito del regulador será complicado poder llegar a este objetivo y obtener modelos confiables [14].

El problema de los sesgos en los sistemas de IA constituye otra preocupación clave en el desarrollo y despliegue de esta tecnología. Estos sesgos emergen principalmente de las características inherentes a los datos de entrenamiento utilizados. Los datos utilizados en los sistemas de IA reflejan los prejuicios históricos, culturales y sociodemográficos presentes en las fuentes de entrenamiento. Esto puede conducir a decisiones médicas que favorezcan o perjudiquen de manera sistemática a ciertos grupos, como géneros, edades o etnias específicas. Por ejemplo, estudios han demostrado que algunos algoritmos clasifican erróneamente condiciones dermatológicas en personas con piel oscura debido a la falta de diversidad en los datos de entrenamiento. La complejidad del problema se magnifica por las dificultades en la “explicabilidad” de las respuestas generadas, donde la identificación precisa de las fuentes de sesgo resulta técnicamente muy complicada. Para mitigar los sesgos en los algoritmos de la IA en salud, los desarrolladores deberían garantizar que cada etapa del proceso sea inclusiva y representativa de la realidad. Un factor fundamental podría ser diversificar las fuentes de datos, integrando información de distintas regiones y subgrupos, para asegurar que los modelos reflejen adecuadamente la diversidad humana [15, 16]. No obstante, algunos autores reconocidos, como Yuval Noah Harari, se posicionan de una manera poco optimista. Para Harari, la problemática inherente a los sesgos en los sistemas de IA trasciende la mera cuestión técnica, sino que constituye una limitación fundamental. Los corpus de entrenamiento utilizados en el desarrollo de estos sistemas están impregnados de sesgos que son ineludibles a la producción del conocimiento humano. La pretensión de desarrollar sistemas de IA libres de sesgos se enfrenta a una paradoja fundamental, requeriría la construcción de un nuevo corpus de conocimiento alternativo completamente desprovisto de sesgos. Esto es una tarea que no solo es inviable desde una perspectiva operativa, sino que probablemente sea conceptualmente imposible, dado que la generación de este nuevo conocimiento tendría los nuevos sesgos establecidos por sus creadores [17].

Ante el problema de los sesgos en la IA, una alternativa es la creación de datos sintéticos para que sirvan de entrenamiento. Los datos sintéticos son conjuntos de datos generados artificialmente que imitan las características de los datos reales. Su utilidad en la eliminación de sesgos radica en la potencial capacidad para equilibrar la participación de grupos infrarrepresentados y corregir desigualdades estructurales en los datos originales. No obstante, su generación es compleja y su eficacia en el entrenamiento efectivo de los sistemas de IA debe demostrarse [18]. Otra opción es el empleo de técnicas de Generación Aumentada por Recuperación (RAG, por sus siglas en inglés *Retrieval-Augmented Generation*). Esta técnica combina la generación de texto por parte del modelo con la recuperación de información de fuentes externas seleccionadas, proporcionando un contexto más específico, actualizado y relevante. El objetivo principal del RAG es complementar el conocimiento pre-entrenado del modelo con datos externos en tiempo real, lo que permite mejorar la precisión de las respuestas y, en algunos casos, facilitar la trazabilidad de las fuentes. El uso de RAG podría mitigar sesgos si las fuentes externas son diversas, equilibradas y verificables, ya que estas permiten contrarrestar posibles limitaciones o errores en los datos pre-entrenados del modelo. No obstante, la eficacia para reducir sesgos dependerá a su vez de la calidad y supervisión de las fuentes utilizadas. Por lo tanto, aunque el RAG es un enfoque interesante, no representa una solución definitiva. Los sistemas que lo implementan siguen enfrentando desafíos similares a los modelos pre-entrenados, como la dependencia de la calidad de los datos utilizados [19].

Aunque puede parecer obvio, es obligatorio que la integración de la IA en medicina deba estar respaldada por los estándares habituales de rigor científico [20, 21]. Las herramientas basadas en IA deben someterse a una evaluación transparente y reproducible, con datos validados por grupos independientes. Solo así se garantizará que su uso cumpla con los criterios de calidad, seguridad y efectividad exigidos en cualquier intervención médica, promoviendo una práctica basada en la evidencia. La normativa para aprobar una innovación basada en IA debe incluir una definición clara del objetivo clínico y una estrategia de gestión de riesgos con el fin de mitigar posibles daños, especialmente en contextos con reducida supervisión profesional. Tampoco debe dejarse de lado la eficiencia de las innovaciones mediante una evaluación de su costo-efectividad y de los recursos necesarios para su implementación.

Una prioridad en el uso de la IA en la asistencia sanitaria es la reducción de las tareas repetitivas y de bajo valor añadido, como la gestión administrativa, la programación de citas o la transcripción de datos clínicos [22, 23, 24]. Estas actividades, aunque necesarias, consumen un tiempo significativo del profesional sanitario que podría destinarse a labores de mayor relevancia clínica, como la toma de decisiones complejas, la comunicación médico-paciente o el diseño de estrategias terapéuticas personalizadas. Sin embargo, el ahorro de tiempo logrado no debe ser canalizado exclusivamente hacia un incremento en la productividad asistencial, como atender a un mayor número de pacientes o acortar los tiempos de consulta. Si se hace así, corremos el riesgo de deshumanizar aún más la relación médico-paciente, un aspecto crítico ya tensionado en muchas áreas de la medicina contemporánea. Por el contrario, es fundamental que los recursos de tiempo liberados por la IA se empleen proactivamente para enriquecer la comunicación y reforzar el vínculo terapéutico. Al priorizar estas actividades, la IA no solo contribuiría a la eficiencia operativa, sino que también actuaría como un catalizador para la humanización de la atención médica.

Los agentes de IA representan un avance significativo en la manera en que interactuamos con la tecnología. Son sistemas diseñados para comprender, razonar y responder a tareas específicas o generales, simulando habilidades humanas. Pueden actuar en diversos ámbitos como asistentes virtuales, *chatbots*, herramientas de automatización o analistas de datos. En la asistencia clínica tenemos una gran oportunidad con el empleo de *chatbots* o agentes conversacionales (AC) interactivos. Estos agentes pueden ofrecer soporte constante, información personalizada y seguimiento a personas con condiciones crónicas. Un estudio reciente que comparaba las respuestas proporcionadas por *ChatGPT* 4.0 frente a expertos humanos demostraron claras ventajas de los AC [25]. Los pacientes calificaron a *ChatGPT* como significativamente más empático y más útil en comparación con las respuestas de los expertos. No obstante, los modelos no están exentos de riesgos asociados a generación de información errónea, por lo que es esencial garantizar alguna forma de supervisión profesional. Además, es fundamental garantizar la transparencia en el proceso de integración de los AC para pacientes y profesionales. Los usuarios deben estar plenamente informados sobre si están interactuando con un AC o con un profesional humano, y tener conocimiento del alcance de la participación de los sistemas de IA en la gestión de su atención sanitaria.

## 4. Impacto de la Inteligencia Artificial en la Investigacion Biomedica

La privacidad y protección de los datos personales representan un valor fundamental en asistencia e investigación biomédica. Es responsabilidad del investigador garantizar que todas las herramientas empleadas respeten estrictamente las normativas de privacidad vigentes, como el Reglamento General de Protección de Datos (RGPD) en Europa. Además, los pacientes y sujetos participantes deben recibir una información clara y detallada sobre las medidas de protección de datos implementadas, así como sobre sus limitaciones. Obviamente, estos riesgos y limitaciones de la IA deberían ser explícitos en los consentimientos informados, en el caso que se vayan a emplear herramientas de este tipo. Por último, como ya hemos visto previamente, los investigadores deben ser conscientes de los posibles sesgos inherentes a los sistemas de IA. Estos sesgos pueden generar resultados discriminatorios o no representativos, afectando la validez y la aplicabilidad de los hallazgos en investigación. Para mitigar este riesgo, es esencial facilitar la reproducibilidad de los estudios de investigación mediante el uso de bancos de datos y herramientas de IA alternativas. Este enfoque no solo fortalece la credibilidad de los resultados, sino que también contribuye al desarrollo de sistemas más equitativos y fiables.

Un aspecto transversal en el uso de la IA es la creación de material para contextos asistenciales, educativos o investigación. La potencialidad de estas herramientas es enorme, aunque algunos estudios muestran que poco más del 11% de los investigadores conocen bien las herramientas de IA generativa [26]. La IA puede ser especialmente útil tanto en la generación de ideas, revisión de literatura, redacción académica y el análisis de datos [27]. Sin embargo, existe el riesgo de plagio y la presencia de errores generados por la IA. Estos errores son conocidos popularmente como “alucinaciones” del modelo. Habitualmente se denominan “alucinaciones” a las respuestas erróneas producidas por modelos de IA generativa, aunque formalmente tengan consistencia narrativa y verosimilitud en una lectura rápida de la misma. En lenguaje médico estaría más cerca del concepto de “fabulación”, aunque la terminología nació desde el ámbito de la ciencia de datos. Habitualmente, estos errores pueden deberse a una combinación de factores, como sesgos en los datos originales, insuficiencia de contexto en las entradas del usuario, o la tendencia de los modelos a completar el texto con patrones estadísticos en lugar de garantizar la precisión. Es importante subrayar que todos los modelos de IA generativa se basan en modelos probabilísticos, y por tanto no exentos de errores. Además, la falta de supervisión explícita durante el entrenamiento puede llevar al modelo a generalizar de manera errónea, generando respuestas que aparentan ser correctas pero que en realidad no lo son. Un problema añadido es la dificultad para tener cifras reales de los errores generados por los modelos de IA generativa, en parte por la rápida evolución de las herramientas, y también por la falta de transparencia de la industria tecnológica. Esto ha generado publicaciones que cifran estos errores entre el 3% y el 27% [28]. Actualmente se tiende a evaluar la precisión de los modelos mediante técnicas como la Puntuación de Coherencia Fáctica (PCF) desarrollada por empresas tecnológicas independientes [29]. La PCF establece un punto de referencia para la detección de alucinaciones en tiempo real y ofrece el rendimiento y velocidad del modelo. Estos datos permiten a las organizaciones implementar la IA reduciendo la exposición a responsabilidades que puedan surgir de las respuestas erróneas [30]. En la última actualización (en el momento de escribir este trabajo en diciembre de 2024), la tasa de PCF se encontraba entre el 1,3% y el 29,9% dependiendo del modelo analizado [31]. Entre los modelos más populares, las tasas oscilaban entre *Google Gemini-2.0-Flash-Exp* con un 1,4%, similar a *GPT-4o* con un 1,5%, seguido de Anthropic Claude-3-5-sonnet con 4,6%, *Google Gemini-1.5-Flash* con 6,6% o Anthropic Claude-3-sonnet con 16,3%. Por ello, el contenido generado por IA siempre debe ser revisado cuidadosamente para garantizar su fiabilidad, integridad y alineación con el objetivo de los autores.

## 5. Propuesta de Uso Responsable de la Inteligencia Artificial Generativa

En el presente trabajo se propone un conjunto de diez directrices fundamentales que pueden actuar como marco orientativo para una implementación responsable, ética y prudente de los sistemas de IA generativa en el ámbito sanitario (Tabla 1). Estas recomendaciones buscan equilibrar la incorporación de herramientas tecnológicas con la preservación de los valores fundamentales de la práctica clínica, velando por la seguridad del paciente y la integridad del acto médico. Un primer punto es el empleo de la IA como herramienta de apoyo clínico, no como sustituto del juicio profesional. Se enfatiza el papel de la IA como herramienta complementaria, destinada a asistir en la toma de decisiones clínicas, pero sin desplazar la autonomía, el criterio clínico ni la responsabilidad legal del profesional sanitario. La delegación ciega en sistemas algorítmicos debe ser evitada, favoreciendo un uso reflexivo que potencie, pero no reemplace, las competencias del clínico. Para este objetivo, es clave la preservación de las competencias clínicas tradicionales. La integración de sistemas inteligentes no debe suponer una merma en las habilidades clínicas básicas como la anamnesis, la exploración física o la capacidad de razonamiento diagnóstico. Es imperativo proteger y cultivar estas destrezas, reconociéndolas como elementos centrales de la relación médico-paciente, cuyo deterioro podría comprometer gravemente la calidad asistencial. Como cualquier innovación en el área médica, es fundamental realizar una evaluación rigurosa de la evidencia científica que apoya cada implementación de IA en medicina. La fascinación que despiertan las innovaciones tecnológicas debe ser moderada mediante una evaluación crítica y sistemática de la evidencia disponible. La IA, al igual que cualquier intervención diagnóstica o terapéutica, debe someterse a los principios del método científico y la medicina basada en la evidencia, incluyendo estudios prospectivos, potencialmente replicables y con un análisis del impacto clínico. Un posicionamiento importante, desde el punto de vista asistencial, es priorizar la aplicación de la IA generativa en la reducción de las tareas administrativas y repetitivas de bajo valor clínico. La IA debería centrarse en la eliminación de cargas administrativas, como la gestión de agendas, el procesamiento de textos o el cribado documental, permitiendo liberar tiempo para actividades clínicas de alto valor, como la atención directa al paciente y la toma de decisiones complejas.

Un aspecto a tener en cuenta en el uso de los sistemas de IA es la trasparencia en su aplicación. Este punto debe ser un principio rector en el diseño, implementación y uso de este tipo de tecnologías inteligentes. Es indispensable que los pacientes, profesionales e instituciones conozcan cuándo y cómo se están utilizando sistemas de IA en su atención o investigación, incluyendo información sobre sus limitaciones, mecanismos de decisión y posible impacto. Obviamente, deberá garantizarse la protección de la privacidad de los datos personales empleados en su entrenamiento y uso clínico. Se debe garantizar el cumplimiento estricto de los marcos legales de protección de datos personales y confidencialidad, como el Reglamento General de Protección de Datos (RGPD) en Europa. Esto implica desde el diseño ético del software (*privacy by design*) hasta la anonimización de datos clínicos y el uso controlado de información sensible.

Como hemos podido describir previamente, uno de los desafíos más relevantes en la aplicación de IA es la presencia de sesgos en los datos de entrenamiento, que pueden derivar en resultados discriminatorios o clínicamente inapropiados. La revisión sistemática de estos sesgos, su monitorización continua y la inclusión de poblaciones diversas en los *datasets* son requisitos indispensables para garantizar la equidad y validez de los modelos. Esta estrategia debe garantizar la reproducibilidad y validación externa de los resultados obtenidos en la aplicación de la IA generativa. Se trata de un pilar clave para garantizar la fiabilidad de los sistemas antes de su adopción generalizada.

Es indispensable que el personal sanitario tenga una formación integral en las aplicaciones, riesgos y limitaciones de la IA. Para ello, ee propone una formación estructurada que abarque desde el pregrado hasta la formación continuada, incluyendo nociones básicas de programación, fundamentos de *machine learning*, pensamiento crítico del uso habitual de la IA y el desarrollo de un marco ético apropiado. Para alcanzar los objetivos anteriores, se requiere de un esfuerzo coordinado entre universidades, administraciones sanitarias y sociedades científicas, orientado a la creación de programas formativos específicos. Esta sinergia permitirá dotar a los profesionales de herramientas adecuadas para interpretar, utilizar y co-crear soluciones basadas en IA dentro de sus respectivas disciplinas.

En cualquier caso, un requisito ético básico debería ser garantizar la transparencia en el uso de estas herramientas. Es fundamental que los autores detallen de forma explícita el grado de implicación de la IA generativa en la generación de contenido. Este autor ofrece una propuesta para comunicar en qué grado se ha empleado herramientas de IA generativa con este objetivo (Tabla 2). Esta graduación se ha realizado basándose en dos aspectos clave. Por un lado, se debe analizar el grado de participación del autor humano frente a la IA en el desarrollo de los contenidos; y, por otro, el nivel de autonomía que ha tenido la IA en todo el proceso creativo. Con estos dos factores se propone una escala en 6 escalones que oscilan entre un escalón 0 donde ni siquiera se emplea un procesador de textos tradicional, hasta un escalón 6 donde la IA genera todo el contenido sin ningún tipo de supervisión humana. Esta información no solo contribuye a la transparencia del proceso, sino que permite valorar la fiabilidad y las posibles limitaciones del material generado. También sería recomendable mencionar la herramienta tecnológica empleada en el desarrollo del contenido; ya que, como hemos visto, el riesgo de errores es variable entre distintos modelos. Independientemente del grado de uso de la IA generativa, el autor debería asumir la responsabilidad última sobre el material desarrollado, asegurando su calidad y precisión, evitando delegar dicha responsabilidad en las herramientas tecnológicas.

**Tabla 2.** **Propuesta de uso transparente de la IA generativa en el desarrollo de contenidos**.

**Escalón 0: Sin asistencia de IA o procesador de texto básico**.
El material es desarrollado exclusivamente por un ser humano sin herramientas tecnológicas. El material es 100% humano.
**Escalón 1: Corrección básica o procesador de texto avanzado**.
El autor se ha ayudado de corrección ortográfica y gramatical, sugerencia de sinónimos o pequeñas modificaciones en frases. Es el uso habitual con procesadores de texto. El material es 100% humano.
**Escalón 2: Mejora estilística y reformulación**.
Se ha empleado La IA ayuda a mejorar la calidad del texto, incluyendo ajustes gramaticales, formulación de frases para mayor claridad o adaptación al tono y estilo deseado. Menos del 10% del material ha sido desarrollado por la IA.
**Escalón 3: Coautoría**.
La IA colabora activamente en el desarrollo del texto, proporcionando ideas o esquemas iniciales, fragmentos redactados para complementar el contenido humano y propuestas creativas bajo instrucciones específicas. La IA ha desarrollado menos del 50% del material, manteniendo el humano la mayor parte de la autoría directa.
**Escalón 4: Creación guiada**.
La IA genera textos completos a partir de instrucciones detalladas que son incluidos en el documento final. La IA puede generar resúmenes de temas complejos o redactar contenido técnico o creativo siguiendo un esquema predefinido por el autor. La IA ha desarrollado más del 50% del material.
**Escalón 5: Creación autónoma supervisada**.
La IA crea textos de manera independiente sin intervención humana directa en el proceso creativo. El autor propone un tema o guion básico y la IA genera contenido técnico, literario o promocional de manera autónoma. El autor puede supervisar y modificar en menor o mayor medida dicho contenido. Menos del 10% del material ha sido desarrollado por el humano.
**Escalón 6: Creación autónoma no supervisada**.
La IA crea material sin intervención humana directa en el proceso creativo y publicada sin supervisión previa o mínima. El contenido es 100% producido por la IA.

## 6. La Formacion en Medicina e Inteligencia Artificial

Todos estos puntos no pueden ser implementados sin una formación estructurada en el campo de la IA a lo largo de todas las etapas de la educación médica. Debe garantizarse un conocimiento sobre el uso, las limitaciones y los riesgos asociados a estas tecnologías con el fin de maximizar su potencial y minimizar posibles consecuencias adversas. Además, como hemos visto, un conocimiento del modelo y funcionamiento de la IA es un elemento importante para poder abordar la “explicabilidad” de las repuestas.

La formación debe abarcar tanto los periodos pregrado como posgrado, asegurando que los futuros médicos y especialistas desarrollen una comprensión crítica y práctica de la IA. En el nivel de pregrado, es imprescindible que los planes de estudio incluyan una introducción formal a los principios fundamentales de la IA, sus aplicaciones en medicina y las implicaciones éticas y legales de su uso. Se debería abordar los principios de la ciencia de datos y el aprendizaje automático y profundo, junto a un enfoque sobre la repercusión ética y seguridad del paciente. Sería aconsejable también tener un conocimiento de las normativas sobre propiedad de datos y de la responsabilidad profesional asociada [32, 33]. En todo caso, parece razonable que esta formación pregrado se ampliara por cada especialidad médica subrayando las aplicaciones en cada campo. Obviamente, esto obligará a reformar múltiples programas e involucrar a todo el claustro de profesores en este cambio de paradigma.

Esta base debería ser complementada con una formación en el posgrado médico. En la situación actual, es razonable que los programas formativos para médicos especialistas incluyan todavía los elementos básicos propuestos para los alumnos de pregrado. Ya hemos comentado que hasta el 50% de los médicos emplean la IA, al menos ocasionalmente, en sus actividades habituales. Sin embargo, también tenemos datos que muestran que tan solo el 24% de los profesionales ha recibido algún tipo de formación específica en este campo. Las limitaciones en la formación incluyen la ausencia de programas de formación certificados, así como la falta de experiencias prácticas que permitan a los médicos familiarizarse con la IA en el contexto clínico diario [1]. Todo ello obliga a desarrollar programas formativos específicos, diseñados para abordar las aplicaciones prácticas de la IA en la clínica, la investigación y la gestión sanitaria. De esta manera, los profesionales podrán integrar esta tecnología como una herramienta de apoyo que complemente su criterio clínico, sin perder de vista los riesgos y las limitaciones inherentes. Para lograrlo, es esencial la colaboración activa entre las administraciones públicas y las sociedades científicas, que desempeñan un papel central en la regulación y el desarrollo de la formación sanitaria. Estas entidades deben trabajar conjuntamente en la construcción de un camino formativo integral y actualizado que actúe como guía para los profesionales sanitarios. Este camino debe estar diseñado para garantizar no solo la adquisición de competencias técnicas, sino también el fortalecimiento de habilidades críticas y éticas que permitan un uso responsable y eficiente de la IA en la práctica médica.

## 7. Conclusiones

La integración de la IA en las ciencias de la salud ofrece un escenario con oportunidades y desafíos, los cuales exigen un abordaje ético, riguroso y equilibrado. Adoptar una postura prudente es una decisión responsable, pero no debe implicar dejar de aprovechar una revolución tecnológica que transformará el mundo en los próximos años. Es fundamental que seamos los propios profesionales de la salud quienes lideremos este cambio, asegurando que la implementación de estas herramientas esté alineada con los valores y principios que sustentan nuestra profesión.

El desarrollo de la IA ha permitido optimizar procesos de diagnóstico, tratamiento y gestión, facilitando la automatización de tareas repetitivas, el análisis de grandes volúmenes de datos y el acceso ágil al conocimiento médico. Herramientas como los grandes modelos de lenguaje, entre ellas ChatGPT, han demostrado utilidad en la generación de contenidos, la asistencia en la toma de decisiones clínicas y la formación médica continua. Sin embargo, estos avances deben ser interpretados con prudencia y sometidos a los mismos estándares de evidencia y rigor científico que cualquier otra innovación en medicina. Actualmente, el uso más consolidado de la IA se encuentra en la automatización de procesos administrativos, la búsqueda de información médica, la generación de contenidos educativos y ciertas tareas de apoyo diagnóstico. A pesar de sus promesas, la IA aún presenta importantes retos en términos de transparencia, “explicabilidad”, equidad y seguridad. La “caja negra” algorítmica, la posibilidad de sesgos en los datos de entrenamiento y la ausencia de regulación específica siguen siendo barreras críticas para su adopción generalizada en un ámbito clínico seguro. En este contexto, el liderazgo profesional resulta esencial para orientar su desarrollo hacia soluciones que estén alineadas con los valores de la medicina centrada en el paciente.

El autor propone un decálogo de directrices esenciales para garantizar una implementación ética, segura y centrada en el profesional sanitario de los sistemas de IA generativa en medicina. Entre los principios destacados, se encuentra la necesidad de mantener la IA como una herramienta de apoyo y no de sustitución del juicio clínico, así como preservar y reforzar las competencias clínicas tradicionales en un entorno cada vez más digitalizado. Del mismo modo, se subraya la responsabilidad última del profesional en las decisiones asistenciales, independientemente del grado de asistencia tecnológica. Otros pilares fundamentales incluyen la priorización del uso de la IA en tareas de bajo valor añadido, promover su integración con criterios de transparencia y respeto a la privacidad, identificar y mitigar los sesgos algorítmicos, y establecer programas formativos específicos que capaciten a los profesionales para un uso crítico y efectivo de estas tecnologías.

La implementación de este conjunto de recomendaciones no solo busca maximizar el potencial de la IA, sino también prevenir la deshumanización del acto médico, asegurando que la tecnología potencie, y no erosione, la relación clínica con el paciente. Nos encontramos en un momento histórico crítico, un punto de inflexión donde las decisiones que tomemos ahora determinarán la trayectoria de la medicina en las próximas décadas. Probablemente, la ventana de oportunidad para establecer un marco adecuado de uso para la IA sea limitada en el tiempo y corra el riesgo de desaparecer si no actuamos con la debida diligencia y determinación. En este momento, una actitud guiada por la inercia o la pasividad podría resultar en un modelo de implementación que no responda a las necesidades reales de pacientes y profesionales sanitarios.

La integración de la IA en el ámbito sanitario representa una transformación significativa en la práctica médica, con un impacto similar a la adopción de la medicina basada en evidencia o la introducción de las pruebas de neuroimagen en neurociencias clínicas. Esta transformación trasciende la mera implementación tecnológica, sino que constituye una reconfiguración fundamental de los procesos asistenciales, formativos y de investigación. La experiencia histórica de la transformación digital iniciada en los años 90, alejada de las necesidades reales de profesionales y pacientes, nos brinda valiosas lecciones sobre la importancia de un enfoque centrado en el usuario final. En el contexto actual, resulta imperativo que seamos los profesionales sanitarios, en estrecha colaboración con los pacientes, quienes lideremos este proceso de transformación. Vivimos una oportunidad histórica para redefinir la práctica médica del futuro, donde la tecnología actúe como catalizador de una medicina más humana, eficiente y centrada en el paciente. Hay que recordar que el futuro de la medicina no está predeterminado por la tecnología, sino por nuestra capacidad para dirigirla hacia los valores fundamentales de la profesión médica.

## Contribuciones de los Autores

DAPM ha realizado la conceptualización, metodología de la revisión, investigación de las fuentes, redacción del manuscrito y revisión del mismo. El autor leyeron y aprobaron el manuscrito final. El autor ha participado lo suficiente en el trabajo y ha acordado ser responsable de todos los aspectos del mismo.

## Aprobación Ética y Consentimiento Informado

No aplicable.

## Agradecimientos

Agradezco los comentarios realizados por el revisor ya que han facilitado la comprensión y clarificación del texto.

## Financiación

Esta investigación no recibió financiación externa.
